# PIK3CA and MAP3K1 alterations imply luminal A status and are associated with clinical benefit from pan-PI3K inhibitor buparlisib and letrozole in ER+ metastatic breast cancer

**DOI:** 10.1038/s41523-019-0126-6

**Published:** 2019-09-23

**Authors:** Mellissa J. Nixon, Luigi Formisano, Ingrid A. Mayer, M. Valeria Estrada, Paula I. González-Ericsson, Steven J. Isakoff, Andrés Forero-Torres, Helen Won, Melinda E. Sanders, David B. Solit, Michael F. Berger, Lewis C. Cantley, Eric P. Winer, Carlos L. Arteaga, Justin M. Balko

**Affiliations:** 1 Department of Medicine, Vanderbilt-Ingram Cancer Center, Vanderbilt University Medical Center, Nashville, TN USA; 2 Breast Cancer Research Program, Vanderbilt-Ingram Cancer Center, Vanderbilt University Medical Center, Nashville, TN USA; 3 Departments of Pathology, Microbiology, and Immunology, Vanderbilt-Ingram Cancer Center, Vanderbilt University Medical Center, Nashville, TN USA; 4 Department of Medicine, Massachusetts General Hospital, Boston, MA USA; 5 University of Alabama, Birmingham, USA; 6 Memorial Sloan Kettering Cancer Center New York, New York, NY USA; 7 Weill Cornell Medical College, New York, NY USA; 8 Dana-Farber Cancer Institute, Boston, MA USA; 9 Harold C. Simmons Cancer Center, UT Southwestern Medical Center, Dallas, TX USA

**Keywords:** Cancer genomics, Cancer genomics, Tumour biomarkers

## Abstract

Clinical trials have demonstrated the efficacy of combining phosphoinositide 3-kinase (PI3K) inhibitors with endocrine therapies in hormone therapy-refractory breast cancer. However, biomarkers of PI3K pathway dependence in ER+ breast cancer have not been fully established. Hotspot mutations in the alpha isoform of PI3K (*PIK3CA*) are frequent in ER+ disease and may identify tumors that respond to PI3K inhibitors. It is unclear whether PIK3CA mutations are the only biomarker to suggest pathway dependence and response to therapy. We performed correlative molecular characterization of primary and metastatic tissue from patients enrolled in a phase Ib study combining buparlisib (NVP-BKM-120), a pan-PI3K inhibitor, with letrozole in ER+, human epidermal growth factor-2 (HER2)-negative, metastatic breast cancer. Activating mutations in *PIK3CA* and inactivating *MAP3K1* mutations marked tumors from patients with clinical benefit (≥6 months of stable disease). Patients harboring mutations in both genes exhibited the greatest likelihood of clinical benefit. In ER+ breast cancer cell lines, siRNA-mediated knockdown of MAP3K1 did not affect the response to buparlisib. In a subset of patients treated with buparlisib or the PI3Kα inhibitor alpelisib each with letrozole where PAM50 analysis was performed, nearly all tumors from patients with clinical benefit had a luminal A subtype. Mutations in MAP3K1 in ER+ breast cancer may be associated with clinical benefit from combined inhibition of PI3K and ER, but we could not ascribe direct biological function therein, suggesting they may be a surrogate for luminal A status. We posit that luminal A tumors may be a target population for this therapeutic combination.

## Introduction

Estrogen receptor-positive (ER+) breast cancer is the most common clinical subtype of breast cancer, and afflicts over 140,000 patients each year in the US.^1^ Although endocrine therapy (aromatase inhibition or selective estrogen receptor modulators/selective estrogen receptor down regulators; SERMs/SERDs) is largely active against ER+ disease, many patients demonstrate or develop therapeutic resistance to endocrine therapy, which can occur through a variety of clinically established mechanisms. Such mechanisms include amplification of *fibroblast growth factor receptor-1* (*FGFR1*), *estrogen receptor-alpha* (*ESR1*) mutations, and aberrant activation of cyclin-dependent-kinase 4/6 (CDK4/6) and PI3K/mTOR. CDK4/6 inhibitors (ribociclib, palbociclib, abemaciclib) are now an approved standard of care in ER+ breast cancer, and their utilization has substantially improved patient outcome. mTOR inhibitors are also approved, but their impact has not been as substantial. Nonetheless, the mechanisms of resistance to endocrine therapy in the clinic are still largely unknown in many of patients.

*PIK3CA*, encoding the p110α subunit of PI3K, is recurrently mutated in 40–50% of ER+ tumors, suggesting a dependency of ER+ breast cancer cells on this pathway.^2–5^ Of these, as many as 80% occur in hotspots within the helical and kinase domains of the p110α isozyme.^2^ It has been well established that these alterations induce PI3K activity resulting in transformation when overexpressed in human mammary epithelial cells, and can induce mammary tumor formation in transgenic mice.^6,7^ Given the role of PI3K in supporting proliferation, survival, and hormone receptor pathway activity, it is not surprising that activation of this pathway is hypothesized to provide cellular avenues to circumvent endocrine therapy sensitivity.

Unfortunately, single agent PI3K inhibitors have not yet demonstrated meaningful activity in clinical trials. Several factors have contributed to their underwhelming performance. First, disruption of the PI3K pathway is followed by feedback activation of compensatory pathways that limit sustained drug target inhibition and suppression of PIP3 production (reviewed in ref. ^8^). One of these adaptive mechanisms is stimulation of C-peptide and insulin secretion, which activates insulin receptors in tumors, further limiting the suppression of PI3K.^9^ Second, therapeutic inhibition with pan-PI3K and PI3K inhibitors is associated with toxicities, such as hyperglycemia, rash, and gastrointestinal side effects as a result of inhibition of wild type PI3K isozymes, hence also preventing sustained and specific inhibition of mutant PIK3CA. Third, mutations in the PI3K pathway are associated with somatic alterations in parallel oncogenic pathways and receptor tyrosine kinases that also stimulate PI3K. Finally, not all clinical trials to date have rigorously selected patients with tumors that are dependent on PI3K signaling and that, therefore, are likely to respond to PI3K antagonists. Early clinical trials suggest PIK3CA mutations are a biomarker of such dependence^10,11^, but that may still not capture the whole repertoire of cancers likely to benefit from these drugs.

Herein, we analyzed next generation sequencing data from a targeted panel of known recurrently mutated genes in cancer in a cohort of patients from two phase Ib trials of buparlisib and alpelisib, each in combination with the aromatase inhibitor letrozole. The goal of this analysis was to determine possible biomarkers aside from *PIK3CA* mutations status that were associated with clinical benefit to the combination. Patients in both trials were endocrine refractory (at least one line of prior failed endocrine therapy), representing an area of therapy that could be greatly impacted by new treatment options. While patients demonstrating clinical benefit (defined as CR/PR or SD ≥ 6 months) were enriched for *PIK3CA* mutations, these alterations were a suboptimal predictor of response. However, the co-presence of alterations in *MAP3K1*, a putative tumor suppressor, with PIK3CA mutations was associated with a significant clinical benefit rate (CBR). This finding spurred molecular analyses to determine if MAP3K1 loss-of-function enhanced PI3K inhibitor sensitivity, which could not be demonstrated. However, we instead found that breast cancers with MAP3K1/PIK3CA co-mutations exhibit a strong luminal A phenotype. Patient-derived xenografts (PDXs) from a variety of solid tumor types showed enhanced responses to both buparlisib and alpelisib when they harbored luminal A-like gene expression. Finally, in both buparlisib and alpelisib-treated ER+ breast cancer patients, luminal A status was associated with a superior CBR. These data suggest that luminal A status and or co-occurring PIK3CA/MAP3K1 mutants may be predictive biomarkers for PI3K inhibition in ER+ breast cancer.

## Results

### Genomic correlates of outcome following buparlisib and letrozole

**Fig. 1:**
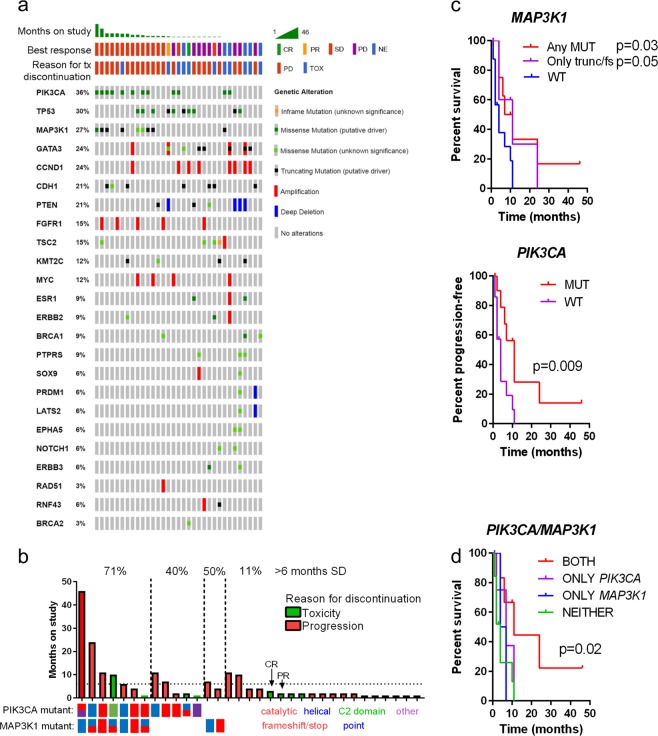
MAP3K1 alterations are associated with improved clinical benefit to PI3Ki and endocrine therapy in PIK3CA^MUT^ ER+ breast cancers. **a** All detected alterations as assayed by IMPACT NGS assay across study patients. **b** Months on study swimmer plot organized by PIK3CA and MAP3K1 mutational status. Patient bars are colored according to reason for discontinuation. **c** Kaplan–Meier progression-free survival curves for patients based on presence/absence of MAP3K1 (top) or PIK3CA (bottom) alterations. Patients who discontinued study medication due to toxicity prior to detection of progression are censored events. *P*-value represents log-rank test. **d** Kaplan–Meier progression-free survival curves for patients based on PIK3CA/MAP3K1 status. Patients harboring alterations in both genes performed significantly better (*p* = 0.02; log-rank trend test), while patients with an alteration in only one of the pathways performed similarly to those with neither

To determine potential genomic correlates with clinical benefit to buparlisib and letrozole in metastatic ER+ breast cancer patients, we performed MSK-IMPACT targeted next generation sequencing (tNGS) profiling for 341 genes using pre-study biopsy (where available) material or archival tissue from 33 patients subsequently enrolled and treated according to the study protocol. Mutations, amplifications, and deep deletions identified by tNGS, organized by time-on-study are shown in Fig. 1a. No genomic lesions were associated with an objective response (partial response PR/CR), as there were only one of each in this cohort. *PTEN* deletions or mutations were associated with PD at first scan, but a deep deletion at the *PTEN* locus was also detected in the only patient to achieve a PR. Interestingly, alterations in *PIK3CA were moderately* enriched in patients with CR/PR/SD versus PD (*p* = 0.09, fisher’s exact) while MAP3K1 alterations were significantly enriched in patients with CR/PR/SD (*p* = 0.01, fisher’s exact). Mutations in *PIK3CA* were primarily hotspot mutations, while *MAP3K1* alterations were primarily truncating or frameshift mutations. The distribution of mutations in both MAP3K1 and PIK3CA are shown in Supplementary Fig. 1.

MAP3K1 is a member of the mitogen-activated protein kinase kinase kinase (MAP3K) family of serine/threonine kinases. The downstream effector of MAP3K1 activity (via MAP2K4) is JNK. Interestingly, MAP3K1 is distinct from most other human kinases in that it simultaneously demonstrates E3-ubiquitin ligase activity which degrades both c-Jun and ERK1/2 via ubiquitination.^12^ MAP3K1 has been shown to play a role in cell migration and survival while cleavage via caspase activation promotes apoptosis. Several genomic studies in breast cancer have revealed that MAP3K1 alterations are most common in the luminal A subtype of breast cancer.^13–16^ The pattern of mutations observed both in our study (Supplementary Fig. 1) and in large-scale genomic efforts^16^ suggest loss-of-function, due to the predominance of truncating and frameshift alterations. Importantly, recent data suggest that MAP3K1 deletion or loss-of-function can activate PI3K through IRS1, though in preclinical studies, this led to resistance, not enhanced sensitivity to PI3K or AKT inhibition.^17^

Next, we examined the association of *PIK3CA* and *MAP3K1* alterations with clinical benefit. CBR tracked with alterations in both genes; 2/5 (40%) of patients with alterations in only *PIK3CA* and 1/2 (50%) patients with only *MAP3K1* mutations derived clinical benefit, whereas 5/7 (71%) patients with tumors harboring both alterations did so. Among these, two patients had SD lasting for 2 years. In contrast, patients with cancers lacking a mutation in either gene had a CBR of only 11%, but both the single CR and PR belonged to this group (Fig. 1b). Both of these patients, despite achieving an objective response, had to come off study prior to 6 months due to toxicity. Since it cannot be determined whether patients who were withdrawn from the study due to toxicity would have continued to benefit or progress, withdrawal from the study for this reason was used as a censor. Using toxicity as censoring variable, stratifying progression-free survival (PFS) by presence vs. absence of a *MAP3K1* alteration or a *PIK3CA* alteration both yielded improved survival (*p* = 0.03 and *p* = 0.009, respectively, log-rank test; Fig. 1c). Importantly, patients harboring truncating or frameshift mutations only, as opposed to point mutations, for which biological consequences are less clear, continued to have improved outcomes (*p* = 0.05; Fig. 1c). However, the longest-responding patient, whose tumor harbored a MAP3K1 L380S mutation, was excluded in this analysis, despite the fact that this is a recurrent mutation in breast cancer (AACR GENIE data v4.1), suggesting it is a loss-of-function mutation. Patients with both PIK3CA and MAP3K1 alterations appeared to achieve the greatest benefit from buparlisib and letrozole relative to patients with an alteration in only one or the other (Fig. 1d).

### PI3K inhibition activates JNK/cJun signaling via MAP3K1

**Fig. 2:**
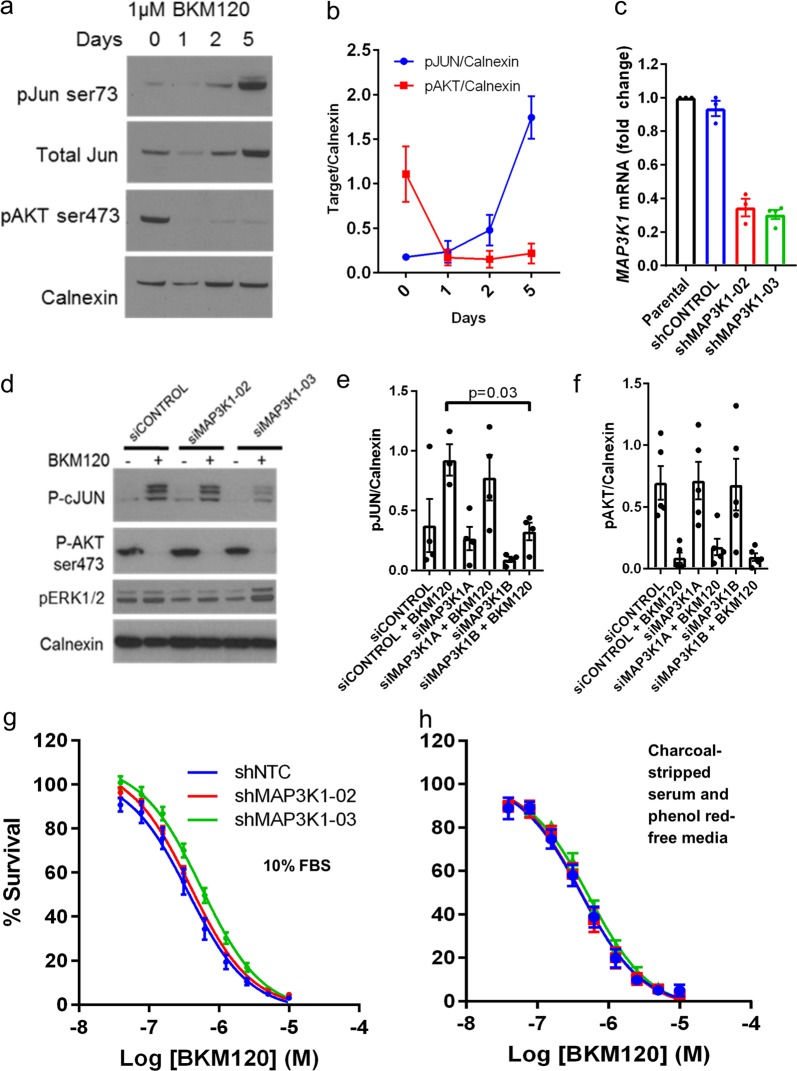
Buparlisib treatment enhances p-cJUN activation as a possible escape mechanism in a MAP3K1-dependent manner, but inhibition of MAP3K1 does not alter buparlisib sensitivity in vitro. **a** T47D cells were treated in full serum media with 1 µM buparlisib (BKM120) for 1, 2, or 5 days. Immunoblotting demonstrated sustained inactivation of p-AKT, but a reciprocal activation of p-cJUN, particularly after 5 days. Images are representative of *n* = 4 independent experiments. **b** Quantification of densitometry of four independent experiments performed similarly to **a**. **c** qPCR analysis of MAP3K1 mRNA in shMAP3K1 and shCONTROL transduced T47D cells. Error bars represent mean ± SEM of four independent experiments. **d** shRNA-transduced T47D cells were treated for 5 days with 1 µM buparlisib followed by immunoblot. Images are representative of *n* = 4 independent experiments. **e** Densitometry quantification p-cJUN of four independent experimental replicates from **d**. Error bars represent mean ± SEM of four independent experiments. *P*-value represents a two-sample two-tailed *T*-test. **f** Densitometry quantification p-AKT of four independent experimental replicates from **d**. Error bars represent mean ± SEM of four independent experiments. **g** Sulfarhodamine B (SRB) assay across dose curve in full serum or estrogen deprived **h** conditions performed at 5 days, normalized to DMSO control for each cell line. Error bars represent mean ± SEM of three independent experiments

We hypothesized that loss of function of MAP3K1 would render ER+ breast cancer cells more sensitive to PI3K inhibition, potential explaining the patient outcome data. The JNK/cJUN pathway, downstream of MAP3K1, is a stress-associated pathway that has been implicated as a therapeutic escape mechanism in cancer cells.^18^ Thus, we first tested the effect of buparlisib on *PIK3CA*^mut^ ER+ T47D breast cancer cells. T47D cells were selected because they express high *MAP3K1* mRNA levels and an activating *PIK3CA* mutation. Of note, we were unable to identify any ER+ breast cancer cell line within the Cancer Cell Line Encyclopedia^19^ with both genomic loss/inactivating mutations in *MAP3K1* coincident with a *PIK3CA* mutation (Supplementary Fig. 2). Treatment with buparlisib demonstrated sustained suppression of S473 p-AKT over a 5-day period in culture. However, as hypothesized, JNK activation measured by S73 p-cJUN was strongly activated in reciprocal fashion (Fig. 2a, b). To determine whether this upregulation of p-cJun was dependent on MAP3K1, we generated T47D cells stably expressing short-hairpin RNA (shRNA) targeting *MAP3K1* (shMAP3K1) or a non-targeting control (shCONTROL). Due to difficulties in identifying effective, reproducible, and robust antibodies to reliably detect MAP3K1 protein, we used qRT-PCR to evaluate knockdown. Approximately 70% mRNA knockdown was achieved with both shRNA sequences used, with the second sequence (shMAP3K1-03) resulting in slightly better suppression (Fig. 2c). MAP3K1 RNA interference demonstrated suppression of p-cJUN induction after 5 days with buparlisib, without affecting baseline p-AKT levels or altering buparlisib-mediated suppression of p-AKT (Fig. 2d–f).

### MAP3K1 loss does not alter sensitivity to PI3K inhibition

**Fig. 3:**
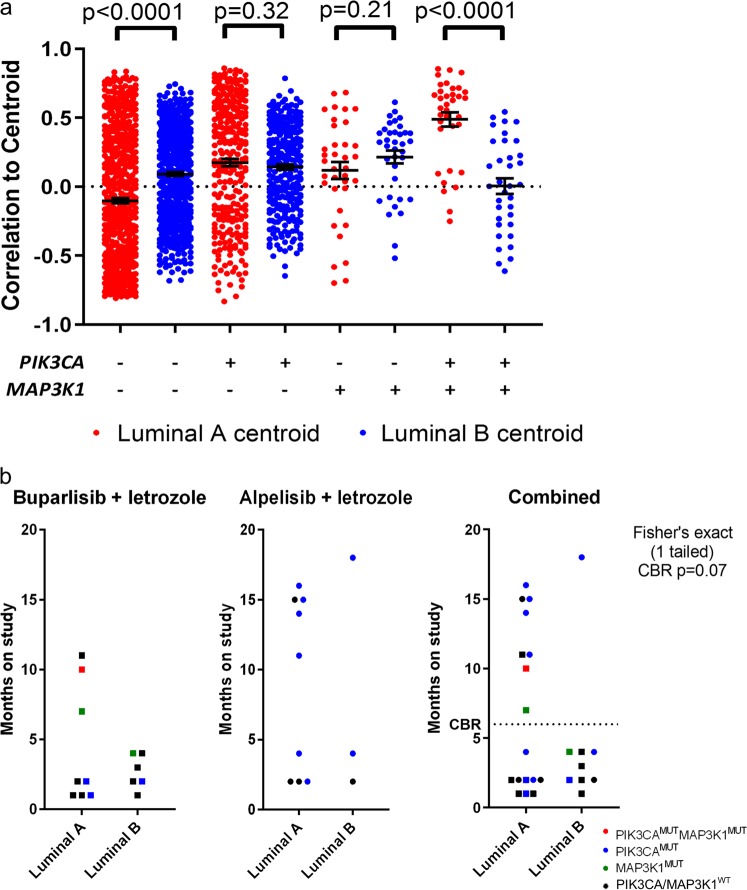
Dual MAP3K1 and PIK3CA alterations are associated with a highly luminal A like phenotype and luminal A status is associated with clinical benefit to PI3Ki with endocrine therapy. **a** RNAseq data from TCGA breast cancers (*n* = 959) were stratified by MAP3K1 and PIK3CA status and used to determine correlation to Luminal A and Luminal B centroids using the genefu package in R. Difference in correlation to both centroids were compared using a paired *T*-test. MAP3K1/PIK3CA altered tumors demonstrated a significantly higher association with Luminal A centroids than Luminal B centroids ***P < 0.0001 by two-sample two-tailed *t*-test. Error bars represent mean ± SEM. **b** PAM50 analysis was performed using a custom NanoString RNA panel on a subset of samples (based on tissue availability) in tumors from patients enrolled in buparlisib + letrozole^25^ or alpelisib + letrozole^21^ (*n* = 14 and *n* = 12, respectively). All samples predicted as luminal A or B. Mutation status is denoted by color of data points. In the combined analysis, luminal A tumors showed a trend for enrichment of patients achieving clinical benefit (CR/PR or SD > 6 months) by a Fisher’s exact test (*p* = 0.07)

Since inhibition of PI3K resulted in cJUN activation, and activation of cJUN was reduced by MAP3K1 loss, we reasoned that loss of MAP3K1 would enhance sensitivity to PI3K inhibitors by eliminating a potential mechanism of adaptation and/or drug resistance. To test this, shMAP3K1 T47D cells were treated across a dose range of buparlisib to determine sensitivity. However, MAP3K1 knockdown did not alter the therapeutic window of buparlisib in T47D in full serum media, or estrogen-deprived conditions (Figs. 2g and 3h, respectively). Additional low density clonogenic assays and cell proliferation in monolayer confirmed this lack of an effect of MAP3K1 loss (Supplementary Fig. 3a). A moderate decrease in shMAP3K1 cell number was observed with buparlisib treatment, but these cells also grew more slowly in the absence of buparlisib (Supplementary Fig. 3b); after controlling for this effect there was no change in buparlisib sensitivity (Supplementary Fig. 3c). Furthermore, transduction of *MAP3K1* siRNA into MCF7, BT483, and T47D cells failed to impact sensitivity to buparlisib in full serum-containing media and in estrogen-deprived conditions (Supplementary Fig. 4). Thus, despite a molecular effect of MAP3K1 loss on cJUN activation following PI3K inhibition, there was insufficient evidence to suggest that cJUN activation promoted escape from PI3K antagonists.

An alternative hypothesis could be that MAP3K1 loss increases activation of the PI3K pathway, resulting in increased dependence on this pathway. However, we did not observe changes in PI3K activation status (p-AKT) with MAP3K1 loss, or during treatment with buparlisib (Figs. 2d and f). Furthermore, in the TCGA breast cancer dataset, tumors with simultaneous loss of MAP3K1 or MAP2K4, its putative downstream effector, and PIK3CA alterations did not demonstrate enhanced transcriptional readouts (PIK3CA-GS, see ref. ^20^) of PI3K pathway activation (Supplementary Fig. 5).

### Luminal A gene expression associates with response to buparlisib and letrozole

Large genomic studies have shown that luminal A tumors are more likely to harbor both *PIK3CA* and *MAP3K1* alterations. Thus, we reasoned that buparlisib may elicit preferential clinical activity against this molecular subtype of breast cancer. We speculated that this could conceivably arise from a differential dependency of more well-differentiated (i.e. luminal A) tumor cells on PI3K signaling compared to tumors that are more de-differentiated (luminal B and others). We first tested this hypothesis using PAM50 analysis of the TCGA breast cancer data (*n* = 959). Using correlation of the expression of each tumor to the luminal A and luminal B PAM50 centroids, we found a strong correlation of tumors with both *MAP3K1* (mutations/deletions) and *PIK3CA* mutations with a luminal A-like expression pattern vs. a luminal B expression (*p* < 0.0001; Fig. 3a). This was unique to the tumors harboring both mutations, and was not identified in PIK3CA-only mutated or *MAP3K1*-only altered tumors. Thus, co-occurrence of *MAP3K1* and *PIK3CA* mutations is associated with a strong luminal A-like phenotype in ER+ breast cancer.

Based on these findings, we isolated RNA from a subset (*n* = 14) of available tumors from patients in the buparlisib trial as well as in 12 tumors from patients in a subsequent study of alpelisib plus letrozole.^21^ Using a custom-designed NanoString codeset targeting the PAM50 geneset, we ascertained the molecular subtype by testing the association to luminal A or luminal B PAM50 centroids. Interestingly, all three patients with clinical benefit to buparlisib/letrozole had a luminal A-like gene expression pattern in their cancer. Of these, only one had mutations in both *MAP3K1* and *PIK3CA*, while one had only a *MAP3K1* alteration and the third had no alterations detected in either gene (Fig. 3b). The alpelisib trial was heavily enriched with patients with *PIK3CA* mutations but contained no patients with *MAP3K1* mutations. Five of six available tumors from patients who derived clinical benefit to alpelisib/letrozole were of luminal A subtype. Overall in the combined analysis of both trials, there was a trend toward significance (*p* = 0.07, 1-tailed Fisher’s exact) for an association of luminal A status with clinical benefit to PI3K inhibition plus estrogen suppression with letrozole. Thus, these data support the hypothesis that a luminal A lineage may predict benefit of PI3K-targeted inhibitors in ER+ disease, but this association should be confirmed in a larger clinical trial.

**Fig. 4:**
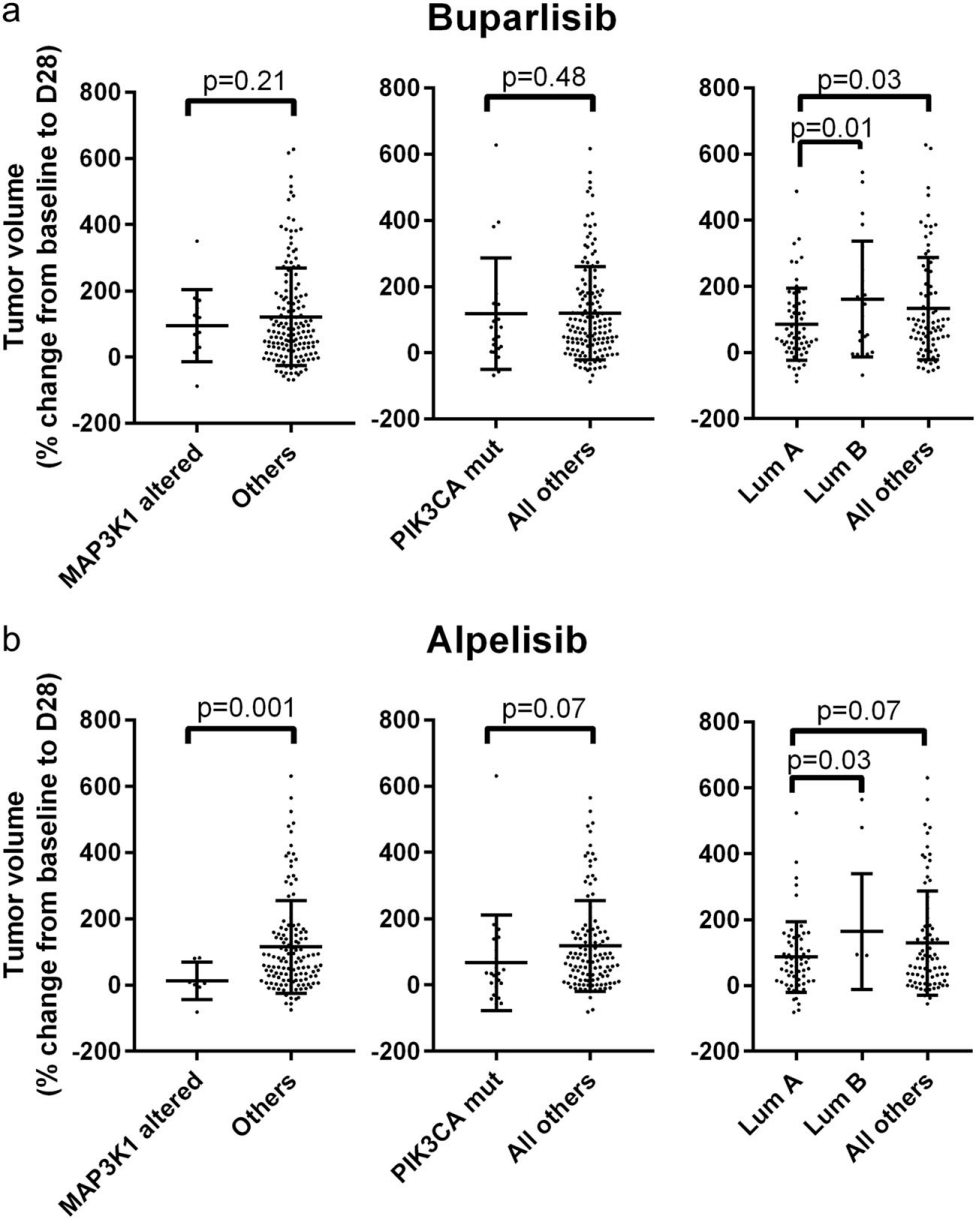
Association of MAP3K1, PIK3CA, and Luminal A status with sensitivity to PI3K inhibition in PDX tumors. **a** Association of buparlisib sensitivity across 170 PDX models, stratified by presence/absence of a *MAP3K1* alteration or *PIK3CA* mutation, or luminal A/B status. *P*-values represent a one-tailed *t*-test, with Welch’s correction for unequal variance. **b** Association of alpelisib sensitivity across 121 PDX models, stratified by presence/absence of a *MAP3K1* alteration or *PIK3CA* mutation, or luminal A/B status. *P*-values represent a one-tailed *t*-test, with Welch’s correction for unequal variance

To provide additional evidence to this end, we leveraged a large database of PDXs^22^ from a variety of tumor types, treated with buparlisib or alpelisib. We asked whether PAM50 subtyping of the PDXs, which has been applied to other tumor types outside of breast cancer,^23,24^ or the presence of genomic alterations in *MAP3K1* or *PIK3CA* were associated with an improved response in vivo to PI3K inhibition. Characteristics of the PDX models analyzed are available as Supplementary Dataset 1. The response (change in tumor volume at day 28 after starting treatment, or last time point if <28 days) in each PDX (*n* = 1 per model per treatment^22^) was stratified by *MAP3K1* alterations (mutations or deletions), *PIK3CA* mutations, or luminal A status versus Luminal B versus all others, as predicted by a scaled-data correlation (RNAseq FPKM) to the PAM50 centroids (Fig. 4). Interestingly, PDXs with *MAP3K1* alterations were more sensitive to alpelisib (one-tailed *p* = 0.001), but not buparlisib. *PIK3CA*-mutant PDX models showed a trend toward an improved response to alpelisib as well (one-tailed *p* = 0.07). However, luminal A status predicted response to both drugs, albeit with a greater number of PDXs harboring luminal A gene expression than having alterations in *PIK3CA* or *MAP3K1*. The PIK3CA-GS^20^ gene signature was also correlated with improved response to both buparlisib and alpelisib (*p* = 0.03 and *p* = 0.02, respectively; Supplementary Fig. 6). Importantly, no endocrine therapy was used in this study, which contrasts with the treatment of patients on the clinical trials. However, in both clinical trials^21,25^ the treatment population was endocrine-refractory tumors, and the argument could be made that the benefit of endocrine therapy on these tumors is unclear. Nonetheless, these data support the notion that luminal A status may be a biomarker for response to PI3K inhibitors, that may also underlie the association of *MAP3K1* alterations with clinical response to buparlisib.

## Discussion

In this study, we explored molecular correlates of clinical benefit in a phase Ib trial of buparlisib and letrozole. We found that genomic markers of luminal A breast cancer were enriched in tumors from patients who derived clinical benefit from the combination. Specifically, those patients with both *MAP3K1* and *PIK3CA* alterations appeared to derive the greatest benefit, over those patients with one mutation or none in either gene. It is important to note that a similar association between *MAP3K1* and *PIK3CA* alterations with outcome on alpelisib were independently identified in a preliminary report from ASCO 2018.^26^ Although there are clear caveats to modeling the molecular effects of loss-of-function of *MAP3K1* with *PIK3CA* mutations in breast cancer cell lines, we did not observe a direct effect of *MAP3K1* loss on PI3K inhibitor sensitivity in vitro. Thus, it is unclear whether MAP3K1 alterations are causal to enhanced sensitivity to PIK3CA antagonists, or if they instead mark a specific tumor phenotype.

Consistent with this second hypothesis, we found that ER+ tumors harboring both MAP3K1 and PIK3CA alterations demonstrate a strong propensity toward a luminal A phenotype. Luminal A like tumors express higher ER levels, are more likely to co-express progesterone receptor (PR) and usually have a low basal proliferative rate. In addition, luminal A like tumors are more sensitive to endocrine therapy, have improved prognosis, and may maintain a more differentiated state. In patient tumors where tissue was available, we compared the gene expression patterns to luminal A/B centroids and found that patients with tumors with a luminal A expression pattern were more likely to derive clinical benefit than patients with luminal B cancers. Furthermore, clinical benefit was observed in patients with luminal A tumors that lacked *PIK3CA* (or *MAP3K1*) mutations. However, it is important to note that these observations were limited to a very small sample size and should be considered anecdotal only.

Several other trials have examined molecular correlates with response to endocrine therapy and PI3K inhibition. Although the FERGI trial, which compared the pan-PI3K inhibitor pictilisib plus fulvestrant to placebo plus fulvestrant in patients with metastatic ER+ was largely negative, patients with PR+ tumors benefitted from pictilisib/fulvestrant.^27^ Since PR+ patients are commonly of the luminal A molecular subtype, these data are consistent with our own. However, in a pre-operative window trial of pictilisib and anastrozole vs. anastrozole alone, treatment with pictilisib preferentially reduced tumor Ki67 levels, a marker of proliferation, in luminal B rather than luminal A cancers.^28^ While these results would appear to oppose those presented in our study, there are likely confounding effects in this analysis; luminal A tumors typically have a very low basal proliferation rate, and are therefore much less likely to demonstrate significant reduction in Ki67 following therapy compared to luminal B tumors. However, it is nonetheless interesting that two separate studies have found luminal A/B phenotypes involved in the response to PI3K inhibitors in ER+ breast cancer.

There are a number of caveats to the current study. First, in large comprehensive genomic studies, such as the TCGA,^16^ MAP3K1 mutations were not associated with loss or reduced mRNA expression. This suggests that one of the following is true: (a) the mutations do not result in nonsense-mediate mRNA decay, but are not translated; (b) the mutations do not result in nonsense-mediate mRNA decay, are translated, but produce non-functional proteins; or (c) there is neomorph function to MAP3K1 mutations in breast cancer that our studies do not recapitulate, which would require more in-depth knock-in mutation studies. Next, we were unable to identify existing cell line models bearing prototypic MAP3K1 mutations. We believe this to be a function of the frequent luminal A status of these tumors and is consistent with the general observation that Luminal A tumors are incompatible with cell line culture, or in many cases, PDX model generation.

There is another possible explanation to the association of *MAP3K1/PIK3CA* alterations with benefit from PI3K inhibition. Luminal A tumors typically have reduced proliferation rates and improved outcomes. Since *MAP3K1* and *PIK3CA* mutations were associated with prolonged stable disease, rather than objective response, it is possible that these tumors were simply growing indolently and, thus appeared to benefit from treatment to a greater degree. A placebo control arm in a randomized trial would be required to address such a concern. Nonetheless, our findings suggest a possible connection of luminal A tumors with response to endocrine therapy combined with a PI3K inhibitor, and support further examination of the breast cancer molecular subtype, as well as MAP3K1 alterations, as a biomarker predictive of response to PI3K inhibitors in large randomized trials with this class of drugs.

## Methods

### Patients

The patients described in this study had histologically confirmed ER-positive/human epidermal growth factor receptor 2-negative metastatic breast cancer refractory to at least one line of endocrine therapy in the metastatic setting or diagnosed with metastatic breast cancer during or within 1 year of adjuvant endocrine therapy. All patients were treated with buparlisib or alpelisib as published previously.^21,25^ Approval was obtained from the ethics committees (IRB# 101057, Vanderbilt University Medical Center) at the participating institutions and regulatory authorities. All patients gave written informed consent. The study followed the Declaration of Helsinki and Good Clinical Practice guidelines.

### Next generation sequencing

Next generation sequencing for 341 genes was performed using the MSK-IMPACT platform on DNA isolated from formalin-fixed, paraffin-embedded surgical, or metastatic biopsies, where available. All samples were procured prior to PI3K inhibitor treatment. The MSK-IMPACT platform and sequencing methods are described elsewhere.^29^ The characteristics of samples used for NGS are presented in Supplementary Table 1.

### Cell culture and inhibitors

All cell lines were purchased from ATCC. MCF7 and T47D cells were maintained in DMEM (GIBCO) supplemented with 10% fetal bovine serum (FBS; GIBCO). BT483 cells were maintained in ATCC-formulated RPMI (ATCC, Catalog no. 30-2001) supplemented with 0.01 mg/ml bovine insulin and 20% FBS. For estrogen withdrawal studies, cells were washed twice in serum-free phenol red-free media prior to addition of fresh phenol red-free DMEM supplemented with 10% charcoal-stripped FBS. NVP-BKM120 was purchased from Selleckchem and solubilized in DMSO at a stock concentration of 10 mM. siRNAs against MAP3K1 were purchased from Ambion and SMARTvector shRNA were purchased from Dharmacon. Lentiviral production was performed in 293FT cells. Cells were transduced and antibiotic selected as previously described.^30^

### Immunoblot analysis

Immunoblot analysis was carried out as described.^31^ Primary antibodies used were: Calnexin (#2433), p-cJUN serine 73 (#3270), cJUN (#9165), ERK (#9102), pAKT serine 473 (#4060), p-S6 (#4858) (all from Cell Signaling. 1:1000), and MAP3K1 (ab220416, Abcam; 1:100). Band intensities were quantitated using ImageJ. All pictured blots derive from the same experiment and were processed in parallel.

### Real-time PCR

Cells were harvested and total RNA was isolated using the Maxwell nucleic isolation machine (ProMega). For real-time PCR analysis, cDNA was synthesized from total RNA using the SensiFast cDNA Synthesis Kit (Bioline). The resulting cDNA was subjected to PCR analysis with gene-specific primers using SsoAdvanced Universal SYBR Green Supermix (BioRad). The housekeeping gene β-Actin was used as the internal control and normalized according to the ddCT method. Primers for MAP3K1 are forward: 5′-tgatgtatggagtgttggctg-3′ and reverse 5′-aatgtgaagggatcgatggag-3′ and β-Actin are forward 5′-agaaaatctggcaccacacc-3′ and reverse 5′-ggggtgttgaaggtctcaaa-3′.

### Cell viability assays

MCF7, T47D, or BT483 cells were plated at a density of 10,000 cells per well in a 96-well plate and treated with a two-fold dilution series of BKM120 for 5 days. Viability was ascertained with sulfarhodamine B (SRB) (ACROS). In brief, cells were fixed with 10% trichloroacetic acid (TCA) at 4 °C for 30 min then stained with 0.4% SRB at room temperature for 10 min. Plates were air-dried, then SRB re-solubilized with 10 mM Tris–HCl, pH 7.5 and quantified by absorbance (490 nm) and normalized to control (DMSO treated). For long-term assays (11 days), cells were carried out in 12-well dishes at a seeding density of 10,000 cells/well. Growth Assays were quantified using a Coulter Counter cell counter. For clonogenic growth assays cells were plated at 1000 cells in a six-well plate. Media and drug were changed every 3 days for 30 days and harvested by fixing with 0.5% crystal violet for 15 min. Monolayer staining intensities were then quantified using a LICOR Odyssey infrared plate reader.

### nCounter PAM50 analysis

NanoString analysis was performed on human tumors using the Elements kit. Briefly, single cross sections of FFPE tumors harvested from patient samples were used for RNA preparation using the Maxwell-16 FFPE RNA kit (ProMega, Madison WI). Fifty nanograms of RNA (>300 nt) were used for input into the nanoString Custom Elements to detect 70 targets corresponding to 50 mRNAs of interest and 20 housekeeping genes. Quality-control measures and normalization of data were performed using the nSolver analysis package and Log-2 transformed data were used to test correlations with PAM50 centroids^32^ using the genefu package in R.^33^ The characteristics of samples used for PAM50 subtyping are presented in Supplementary Table 1. Normalized linear count data for the 50 genes are available in Supplementary Dataset 2.

### Publicly available datasets and gene set analysis

#### CCLE analysis

Data for cell lines was obtained from the Cancer Cell Line Encyclopedia assessed through www.cbioportal.org.^19^

#### TCGA analysis

RNA sequencing FPKM level 3 data were downloaded from the TCGA breast cancer data portal.^16^ Log-2-transformed data were used to test correlations with PAM50 centroids^32^ using the genefu package in R.^33^ Data for mutational analysis of MAP3K1 or PIK3CA in both cell lines were downloaded from the cBio portal.^34^

#### PIK3CA-GS analysis

TCGA mutational data and RNA sequencing data was used to determine the PIK3CA-GS score as previously described.^20^ Briefly, the *z* score of the PIK3CA-GS score (the sum of the expression levels of the genes up-regulated in the mutant tumors minus the sum of the expression levels of the genes up-regulated in WT tumors (*n* = 277 genes)) was used to compare between mutational groups.

#### Patient-derived xenograft inhibitor data

PDXs and drug treatment data were accessed from the Supplementary data file of the report.^22^ PDXs with mutational status and drug sensitivity were compared across genotypes (*MAP3K1* mutations/deletions or *PIK3CA* mutations, excluding truncation mutations). The change in tumor volume at day 28 for each model treated with buparlisib or alpelisib was used, or the last time point if earlier than day 28. RNA FPKM data were used to predict PAM50 subtype, using the pam50.scale model in the genefu R package.

### Statistics

Statistical analysis was performed in R (https://CRAN.R-project.org) and Graphpad Prism.

### Reporting summary

Further information on research design is available in the Nature Research Reporting Summary.

## Supplementary information

Data Set 1

Data Set 2

Supplementary Information

Reporting Summary Checklist

## Data Availability

The data generated and analyzed during this study are described in the following data record: 10.6084/m9.figshare.9700373.^35^ Datasets in Prism file format (and accompanying.txt file versions of these datasets) supporting Figs. 1–4 of this published article, are publicly available in the figshare repository at 10.6084/m9.figshare.9700373.^35^ Next-generation sequencing MSK-IMPACT data generated during this study, are publicly available in cBioPortal at https://identifiers.org/cbioportal:brca_mskcc_2019. NanoString normalized linear count data generated in this study are publicly available in Supplementary dataset 2 of the published article. The datasets analyzed during this study are publicly available in cBioPortal at https://identifiers.org/cbioportal:cellline_ccle_broad (CCLE analysis) and at https://identifiers.org/cbioportal:brca_tcga_pub (TCGA analysis and PIK3CA-GS analysis). Patient-derived xenograft and drug treatment data analyzed in this study are publicly available in supplementary dataset 1 of the published article.
