# Supplementary material for: PIK3CA and MAP3K1 alterations imply luminal A status and are associated with clinical benefit from pan-PI3K inhibitor buparlisib and letrozole in ER+ metastatic breast cancer
Source: NPJ Breast Cancer. 2019 Sep 23;5:31. doi: 10.1038/s41523-019-0126-6 (PMC6757060; doi:10.1038/s41523-019-0126-6)

Supplementary Information for:

PIK3CA and MAP3K1 alterations imply luminal A status and are associated with clinical benefit from pan-PI3K inhibitor buparlisib and letrozole in ER+ metastatic breast cancer

Mellissa J. Nixon[1], Luigi Formisano[1], Ingrid A. Mayer[1,2], M. Valeria Estrada[2,3], Paula I. González-Ericsson[2,3], Steven J. Isakoff[4], Andrés Forero-Torres[5], Helen Won[6], Melinda E. Sanders[2,3], David Solit[6], Michael Berger[6], Lewis C. Cantley[7], Eric Winer[8], Carlos L. Arteaga[9], Justin M. Balko[1,2].

Author Affiliations: Department of Medicine[1], Breast Cancer Research Program[2], Departments of Pathology, Microbiology, and Immunology[3], Vanderbilt-Ingram Cancer Center, Vanderbilt University Medical Center, Nashville, TN, USA; Department of Medicine, Massachusetts General Hospital, Boston, MA, USA [4]; University of Alabama, Birmingham, USA[5]; Memorial Sloan Kettering Cancer Center New York, NY, USA[6]; Weill Cornell Medical College, New York, NY, USA[7]; Dana-Farber Cancer Institute; Boston, MA, USA [8]; Harold C. Simmons Cancer Center, UT Southwestern Medical Center, Dallas, TX, USA[9].

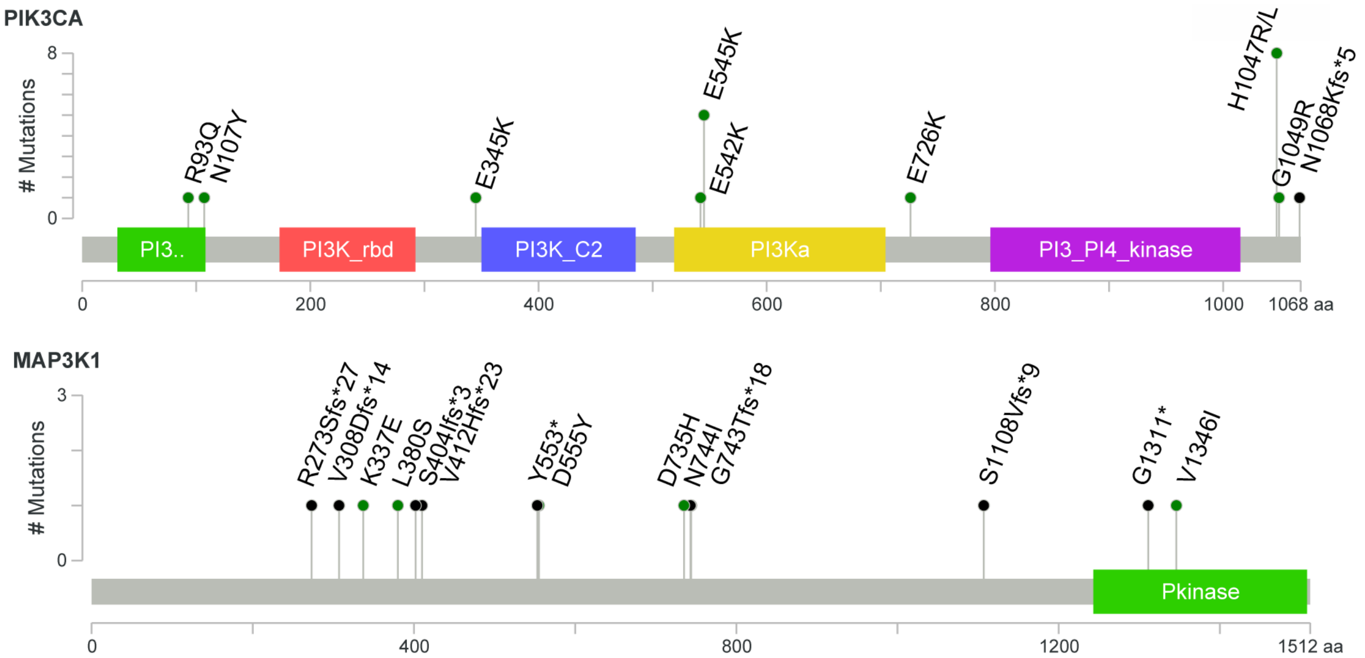

Supplementary Figure 1: Distribution of mutations in PIK3CA and MAP3K1. Lollipop plot reflecting distribution of mutations in *PIK3CA* and *MAP3K1*. Some patients had multiple mutations in the same gene.

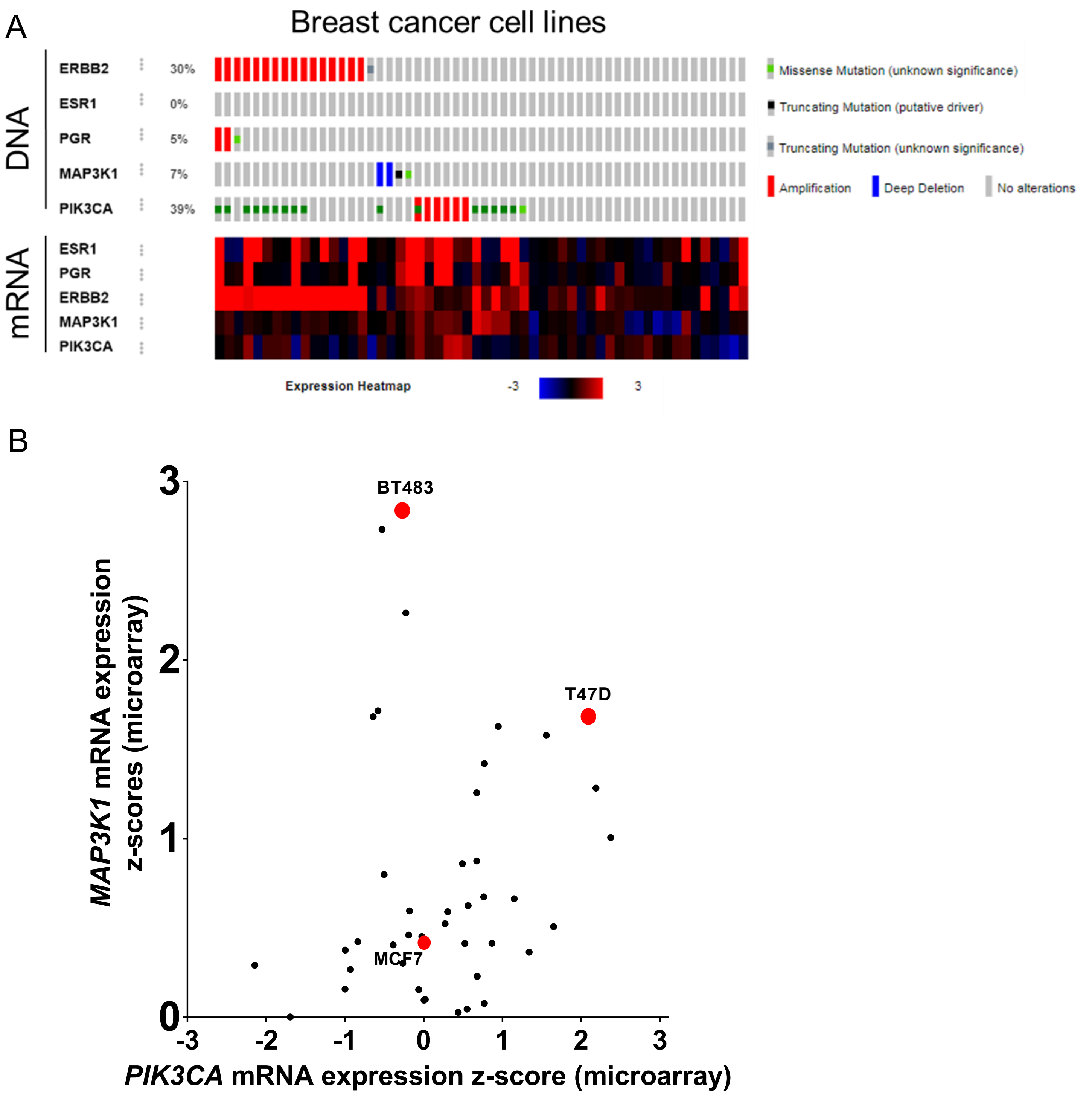

Supplementary Figure 2: Breast cancer cell lines according to PI3K and MAP3K1/MAP3K4 status. A) Oncoprint analysis demonstrating alteration types in candidate genes across CCLE breast cancer cells. B) mRNA expression of MAP3K1 and PIK3CA across CCLE breast cancer cell lines. Cell line models used in this study are identified in red.

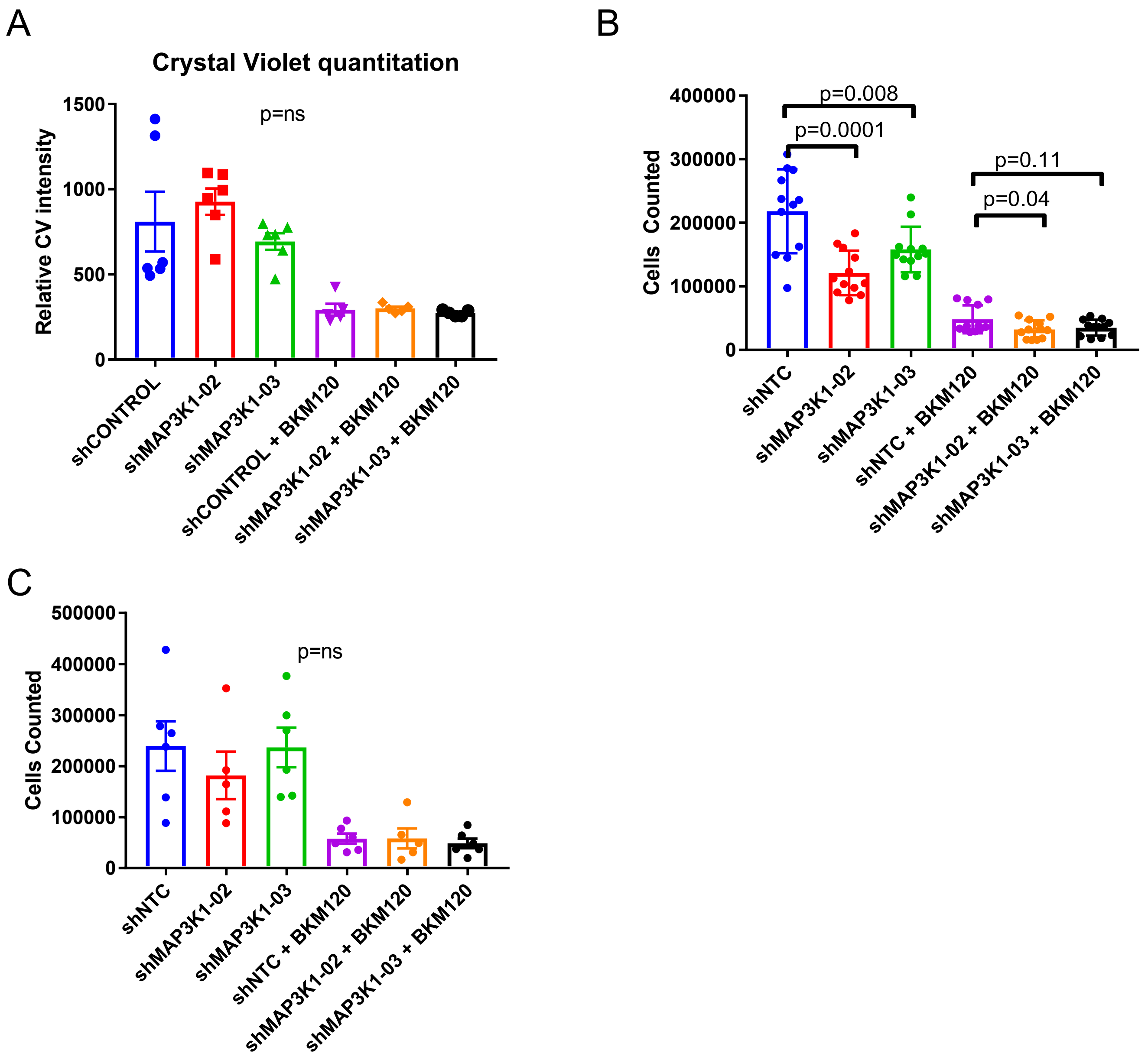

Supplementary Figure 3: RNAi for MAP3K1 does not alter sensitivity to buparlisib in vitro. A) Crystal violet quantification at 12 days post cell seeding of shCONTROL or shMAP3K1 T47D cells treated with DMSO control or buparlisib (1μM). Six independent experiments were performed. B) Direct cell counting (coulter counter) in a similarly designed experiment to (A). shMAP3K1 cells grew slower than shCONTROL cells. Fewer cells remained after buparlisib treatment, but this effect was not conserved after normalizing for basal cell line proliferation, as depicted in (C). P values represent Tukey's contrasts following a significant ANOVA.

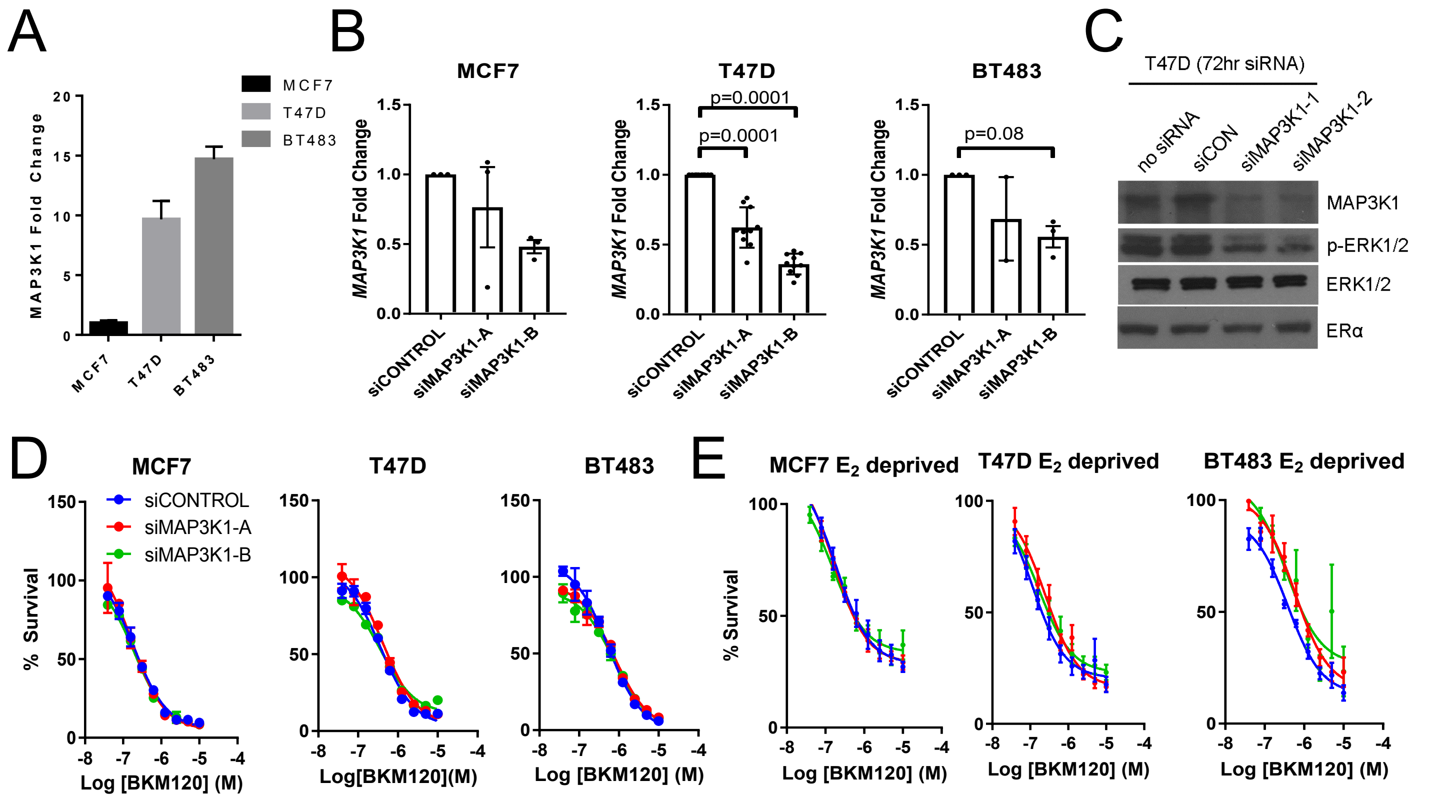

<u>*Supplementary Figure 4: siRNA knockdown of MAP3K1 does not alter sensitivity to buparlisib in vitro.*</u> A) Relative mRNA for *MAP3K1* (normalized to *β-actin*) among ER+ $PIK3CA^{MUT}$ breast cancer cell lines used in this study. B) qPCR analysis of MAP3K1 siRNA knockdown across cell lines at 72 hrs post-transfection. C) Western blot analysis of MAP3K1 knockdown in T47D cells. D) Cell viability at 5 days measured by sulfarhodamine B in cell lines treated across a dose range of buparlisib in normal growth (10% FBS) or (E) estrogen-deprived (charcoal stripped serum and phenol red-free media).

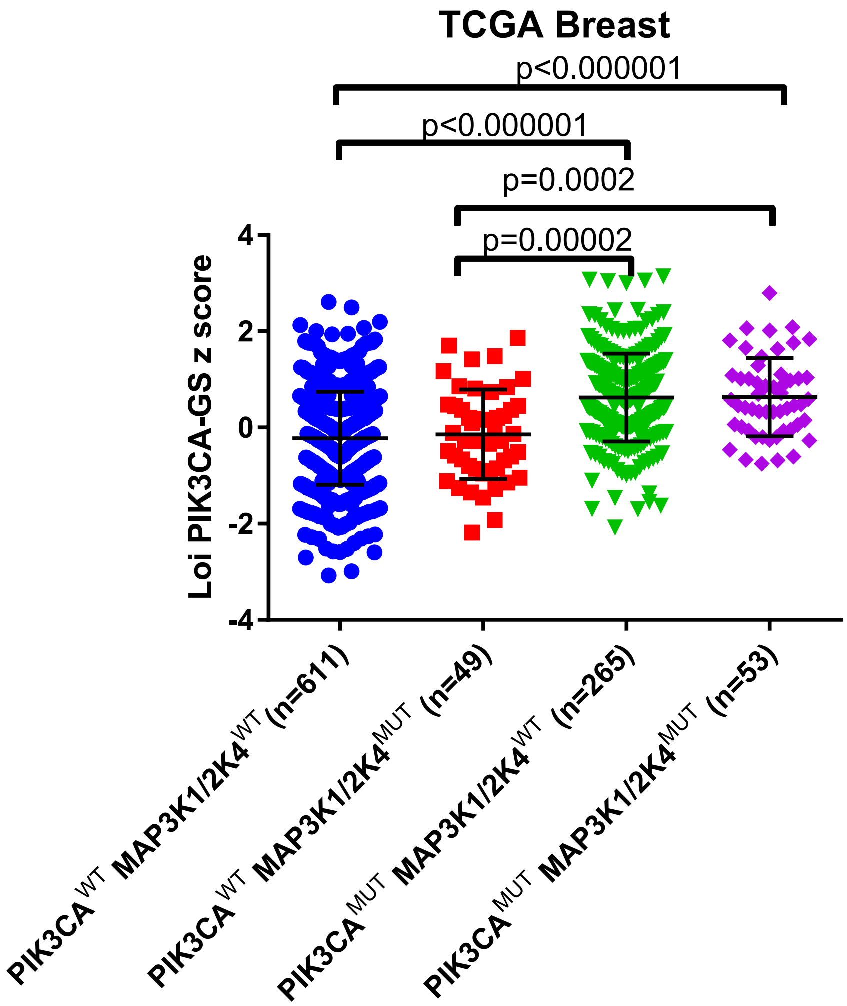

Supplementary Figure 5: MAP3K1 and MAP2K4 alterations are not associated with transcriptional signatures of PI3K activity. TCGA breast cancer RNAseq data were used to calculate the PIK3CA-GS signature as previously described[20] and plotted against presence of *PIK3CA* mutations or mutations in *MAP3K1*/*MAP2K4.* No increase in PIK3CA-GS score was noted in *MAP3K1/MAP2K4* altered specimens, irrespective of *PIK3CA* mutation.

Supplemental Figure 6

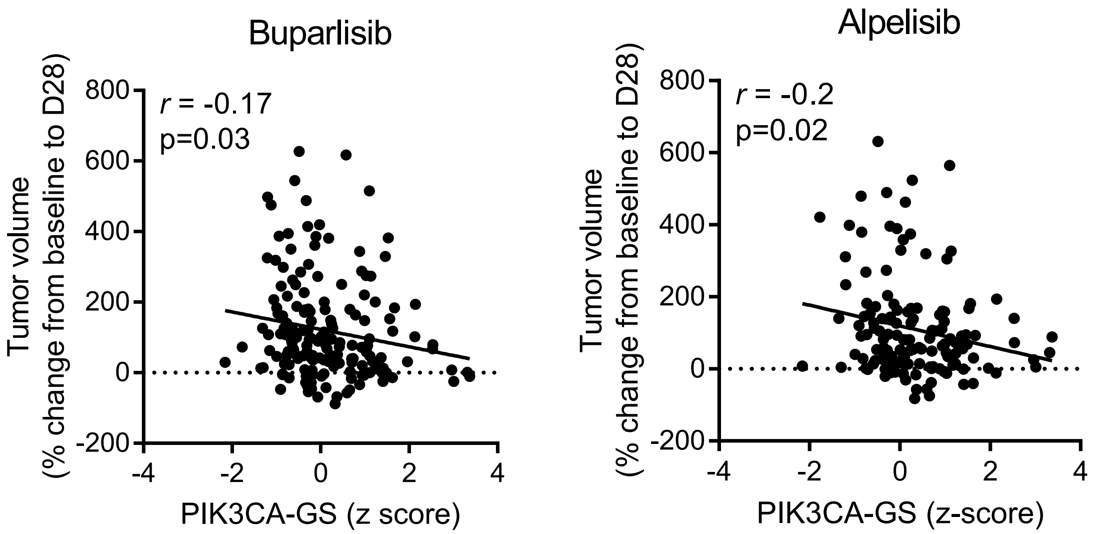

Supplementary Figure 6: Association of PIK3CA-GS with buparlisib and alpelisib response in PDX models. The PIK3CA-GS score was calculated from publicly available RNAseq data from PDX models as described[22], converted to a Z score, and tested for linear trend to changes in tumor volume at day 28 of treatment with the indicated agent, compared to baseline tumor volume (day 0).

Supplementary Table 1:

Study	NGS ID	Dosing Schedule	Sample type	PAM50	Months on study	Best response	Clinical benefit
Alpelisib/BYL719	p_DS_bkm_068	Continuous	Metastasis	luminalA	11	Partial Response	Yes
Alpelisib/BYL719	p_DS_bkm_072	Continuous	Primary	luminalA	2	Progressive Disease	No
Alpelisib/BYL719	p_DS_bkm_074	Continuous	Primary	luminalA	15	Partial Response	Yes
Alpelisib/BYL719	p_DS_bkm_063	Continuous	Metastasis	luminalA	15	Partial Response	Yes
Alpelisib/BYL719	p_DS_bkm_064	Continuous	Primary	luminalA	4	Stable Disease	No
Alpelisib/BYL719	p_DS_bkm_066	Continuous	Primary	luminalA	2	Progressive Disease	No
Alpelisib/BYL719	p_DS_bkm_083	Continuous	Metastasis	luminalA	14	Stable Disease	Yes
Alpelisib/BYL719	p_DS_bkm_062	Continuous	Primary	luminalA	16	Partial Response	Yes
Alpelisib/BYL719	p_DS_bkm_079	Continuous	Primary	luminalA	2	Stable Disease	No
Alpelisib/BYL719	p_DS_bkm_071	Continuous	Primary	luminalB	18	Partial Response	Yes
Alpelisib/BYL719	p_DS_bkm_077	Continuous	Primary	luminalB	4	Stable Disease	No
Alpelisib/BYL719	p_DS_bkm_075	Continuous	Primary	luminalB	2	Progressive Disease	No
Buparlisib/BKM120	p_DS_bkm_030	Intermittent	Primary	NA	46	Stable Disease	Yes
Buparlisib/BKM120	p_DS_bkm_042	Intermittent	Primary	NA	24	Stable Disease	Yes
Buparlisib/BKM120	p_DS_bkm_007	Continuous	Primary	NA	11	Stable Disease	Yes
Buparlisib/BKM120	p_DS_bkm_021	Intermittent	Primary	NA	11	Stable Disease	Yes
Buparlisib/BKM120	p_DS_bkm_001	Continuous	Primary	luminalA	11	Stable Disease	Yes
Buparlisib/BKM120	p_DS_bkm_006	Continuous	Primary	luminalA	10	Stable Disease	Yes
Buparlisib/BKM120	p_DS_bkm_039	Intermittent	Primary	NA	10	Stable Disease	Yes
Buparlisib/BKM120	p_DS_bkm_022	Intermittent	Primary	NA	7	Stable Disease	Yes
Buparlisib/BKM120	p_DS_bkm_013	Continuous	Primary	luminalA	7	Stable Disease	Yes
Buparlisib/BKM120	p_DS_bkm_008	Continuous	Metastasis	NA	6	Stable Disease	Yes
Buparlisib/BKM120	p_DS_bkm_031	Intermittent	Primary	luminalB	4	Stable Disease	No
Buparlisib/BKM120	p_DS_bkm_038	Intermittent	Primary	NA	4	Stable Disease	No
Buparlisib/BKM120	p_DS_bkm_043	Intermittent	Primary	NA	4	Stable Disease	No
Buparlisib/BKM120	p_DS_bkm_002	Continuous	Primary	luminalB	4	Stable Disease	No
Buparlisib/BKM120	p_DS_bkm_003	Continuous	Metastasis	luminalB	3	Partial Response	Yes
Buparlisib/BKM120	p_DS_bkm_005	Continuous	Metastasis	luminalB	2	Complete Response	Yes
Buparlisib/BKM120	p_DS_bkm_009	Continuous	Metastasis	NA	2	Stable Disease	No
Buparlisib/BKM120	p_DS_bkm_017	Intermittent	Metastasis	luminalA	2	Not Evaluable	No
Buparlisib/BKM120	p_DS_bkm_024	Intermittent	Primary	luminalB	2	Progressive Disease	No
Buparlisib/BKM120	p_DS_bkm_025	Intermittent	Primary	NA	2	Progressive Disease	No
Buparlisib/BKM120	p_DS_bkm_029	Intermittent	Primary	luminalA	2	Stable Disease	No
Buparlisib/BKM120	p_DS_bkm_032	Intermittent	Metastasis	NA	2	Progressive Disease	No
Buparlisib/BKM120	p_DS_bkm_034	Intermittent	Primary	NA	2	Progressive Disease	No
Buparlisib/BKM120	p_DS_bkm_035	Intermittent	Metastasis	NA	2	Progressive Disease	No
Buparlisib/BKM120	p_DS_bkm_028	Intermittent	Primary	NA	2	Progressive Disease	No
Buparlisib/BKM120	p_DS_bkm_004	Continuous	Metastasis	NA	1	Not Evaluable	No
Buparlisib/BKM120	p_DS_bkm_010	Continuous	Metastasis	luminalB	1	Progressive Disease	No

Buparlisib/BKM120	p_DS_bkm_018	Intermittent	Primary	luminalA	1	Progressive Disease	No
Buparlisib/BKM120	p_DS_bkm_020	Intermittent	Metastasis	NA	1	Progressive Disease	No
Buparlisib/BKM120	p_DS_bkm_023	Intermittent	Primary	NA	1	Not Evaluable	No
Buparlisib/BKM120	p_DS_bkm_026	Intermittent	Metastasis	luminalA	1	Not Evaluable	No
Buparlisib/BKM120	p_DS_bkm_027	Intermittent	Primary	luminalA	1	Not Evaluable	No
Buparlisib/BKM120	p_DS_bkm_036	Intermittent	Primary	NA	1	Not Evaluable	No

Supplementary Dataset 1: Characteristics and response variables of PDX models analyzed. All data were extracted from[22] and are annotated here for transparency

Supplementary Dataset 2: NanoString normalized linear count data for PAM50 by sample. A custom 60-gene NanoString Elements panel was generated and 500ng RNA was used to examine gene expression patterns for the 50 genes. PAM50 analysis was performed on these data using the Log2 transformed values and no scaling using the genefu package in R.

Uncropped westerns from Figure 3D

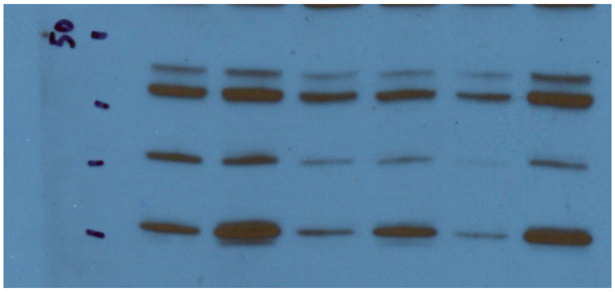

P-ERK

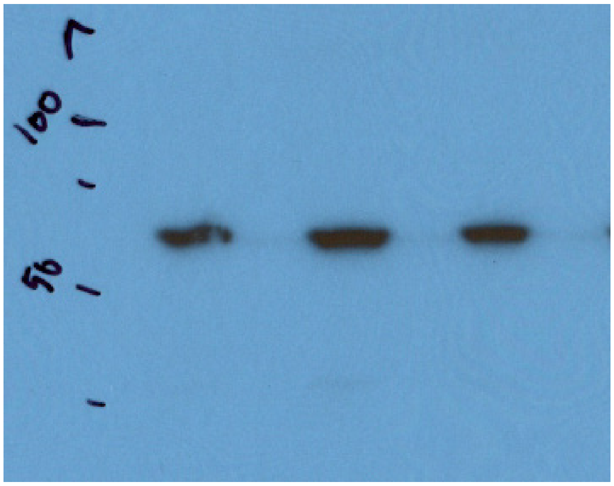

P-AKT

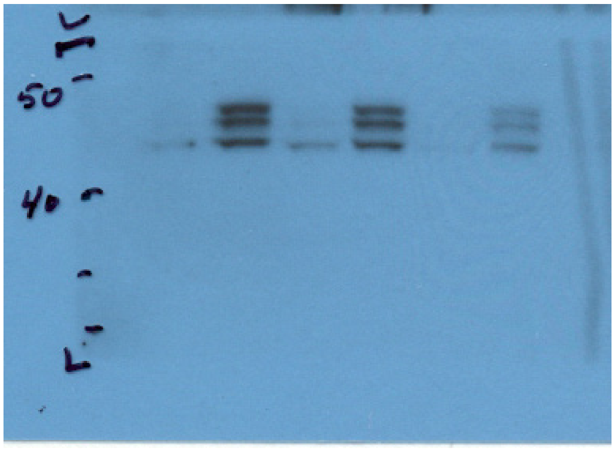

P-cJUN

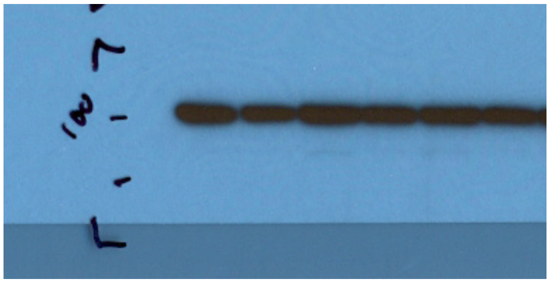

calnexin

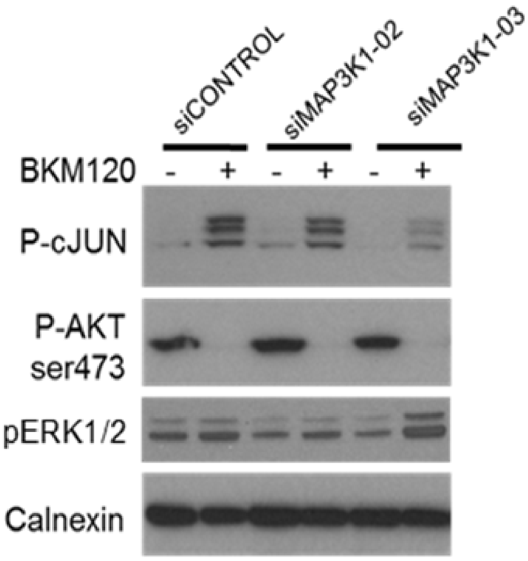

Uncropped westerns from Supplementary Figure 4C

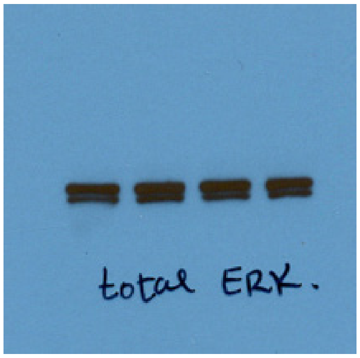

t-ERK

Markers not identified on film

– commonly used, very clean antibody

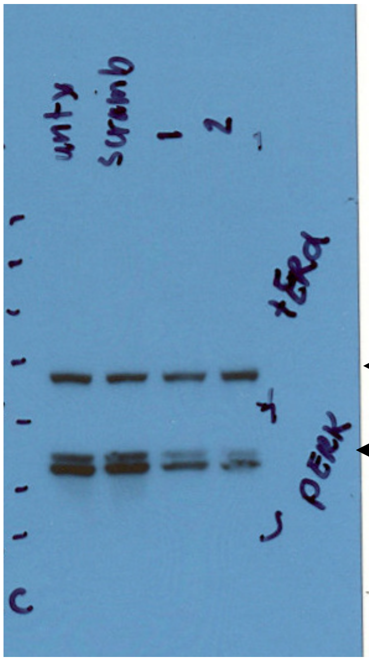

ERα

P-ERK

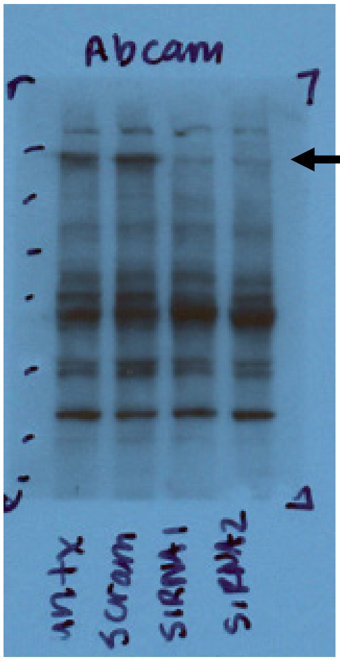

MAP3K1

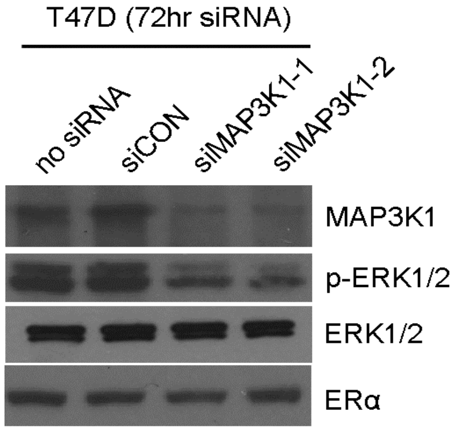

Supplement: Supplementary file 3 — Supplementary Information [file 41523_2019_126_MOESM3_ESM.pdf]
